# Metabolomic Characterization of Pigmented and Non-Pigmented Potato Cultivars Using a Joint and Individual Variation Explained (JIVE)

**DOI:** 10.3390/foods11121708

**Published:** 2022-06-10

**Authors:** Adriana Teresa Ceci, Pietro Franceschi, Enrico Serni, Daniele Perenzoni, Michael Oberhuber, Peter Robatscher, Fulvio Mattivi

**Affiliations:** 1 Department of Cellular, Computational and Integrative Biology (CIBIO), University of Trento, Via Sommarive 9, Povo, 38123 Trento, Italy; adrianateresa.ceci@unitn.it (A.T.C.); fulvio.mattivi@unitn.it (F.M.); 2 Laimburg Research Centre, Laimburg 6, Pfatten (Vadena), 39040 Auer, Italy; enrico.serni@hotmail.it (E.S.); Michael.Oberhuber@laimburg.it (M.O.); 3 Research and Innovation Centre, Edmund Mach Foundation, Via E. Mach 1, 38098 San Michele all’Adige, Italy; pietro.franceschi@fmach.it (P.F.); daniele.perenzoni@fmach.it (D.P.)

**Keywords:** metabolomics, potatoes (*Solanum tuberosum* L.), metabolic characterization

## Abstract

Potatoes (*Solanum tuberosum* L.) are one of the most valuable agricultural crops, and the flesh of these tubers provides various classes of healthy compounds important for human nutrition. This work presents the results of a joint analysis of different chemical classes of compounds which provided insights on the metabolic characterization of pigmented and non-pigmented potato varieties collected from Italy. The identification of common or individual metabolic characteristics across the omic datasets (antioxidants, total polyphenolic content, polyphenols, and sugars) is conducted by Joint and Individual Variation Explained (JIVE), a data fusion multivariate approach. The common part of the multivariate model allowed the separation between non-pigmented and pigmented samples. Polyphenolic compounds were mainly responsible for the separation between purple-fleshed and red-skinned potatoes. An additional detailed analysis of the anthocyanin composition, including the acylated anthocyanins, allowed to pinpoint the diversities between the pigmented potato groups. Furthermore, the presence of an appreciable amount of hydroxycinnamic acids and anthocyanins in the purple-fleshed varieties, which are also characterized by a lower content of sugars, is found. Our results provide scientific evidence for the promotion of promising potato cultivars, which are characterized by a remarkable amount of various health benefit compounds.

## 1. Introduction

According to FAO (Food and Agriculture Organization), potatoes are the most commonly food produced, after maize, wheat, and rice [1]. To date, FAO estimates that 160 countries cultivate potatoes, 4000 varieties are known, and 368 million tons are produced globally. The nutritional potential of potatoes is high because they are rich in various classes of valuable compounds (carbohydrates, proteins, fibers, vitamins, and microelements) and can also contain phenolic acids and flavonoids [2]. However, since their glycaemic index (GI) can be high (it covers a range from 23 to 111), their consumption should be controlled in the presence of specific pathological states like diabetes [3]. Despite this, the American Diabetes Association (ADA) advises to include them in a healthful meal plan [4], suggesting that diabetic people may consider implementing in their daily diet the intake of potatoes characterized by low-GI values. Amongst the different potato varieties, pigmented-fleshed potatoes have been the subject of increasing interest in the last few years showing considerable amounts of antioxidant compounds [5]. Their GI value is indeed reported to be lower compared with non-pigmented potatoes (yellow- and white-fleshed) [6], and the contemporary presence of anthocyanins potentially increases their nutritional value [6,7]. It has been demonstrated that the presence of anthocyanins, combined with other phenolic compounds, may contribute to lowering the glycemic peak after consuming a meal of potatoes [6,7] by inhibiting the intestinal α-glucosidase. In particular, Jokioja et al. [7] have shown that a cooked yellow-fleshed potato portion enriched with purple-fleshed potato extract (rich in acylated anthocyanins and hydroxycinnamic acid derivatives), lowered postprandial glycemic response. This evidence is clearly suggesting that several metabolic features are concurring in defining the nutritional quality of the different potato varieties. To date, however, the literature exclusively reports studies which focus on specific chemical classes, and their results cannot be used for a wide range systematic characterization of the potato metabolome. In this paper, we present the results of an extensive metabolic characterization of 28 cooked potato samples, belonging to 18 different varieties, where a multiclass chemical characterization (total polyphenols, antioxidants, single phenolic compounds, and sugars) was used for a deeper and more comprehensive characterization of the potato metabolome. Following a classification proposed in the literature [2,5,8,9], the samples were organized in four macro groups: yellow-, white-fleshed (non-pigmented), red-skinned, and purple-fleshed (pigmented) potatoes. This broad characterization was complemented by a detailed characterization of the anthocyanins in the pigmented potato samples. Since potatoes are almost always consumed after cooking, we focused on steam cooked samples. Steaming was selected since it was able to retain the maximum amount of bioactive metabolites [10] while limiting in respect to deep-fried potato products the formation of metabolites causing concern, such as Maillard reaction products [11]. In order to investigate the association between the metabolic characteristics, the different datasets were jointly analyzed by a data fusion approach based on Joint and Individual Variation Explained (JIVE) [12]. This type of analysis complemented the characterization of the individual compounds and allowed us to assess the multivariate patterns in the whole dataset, highlighting characteristics that are shared or specifically associated to the different datasets. It is well-known that different degrees of pigmentation resulted in specific coloration patterns in the potato fleshes [2,5,8,9]. It might be the most evident phenotypic difference, however, there were larger genetic differences than the synthesis of pigments [13], thus translating in several types of potato metabolomes. The results of our analysis showed that differences between pigmented and non-pigmented potato varieties extended across the datasets, clearly indicating that the varietal differences in the capacity of synthesizing metabolites had a profound effect on the potato metabolism. At odds, the separation between red-skinned and purple-fleshed varieties was concentrated at the levels of the polyphenols. The in-depth characterization of the pigmented varieties highlighted the presence of specific patterns in anthocyanin composition which determines a clear separation between the different groups. We believe that the proposed approach could be useful to promote the selection of promising potato varieties to increase the awareness of the consumers in food selection.

## 2. Material and Methods

### 2.1. Reagents

Formic acid (LC-MS grade) was obtained from Merck KGaA (Darmstadt, Germany). Acetonitrile (LC-MS grade) and methanol (LC-MS grade) were purchased from VWR International (Radnor, PA, USA). Sodium fluoride, phosphoric acid, glacial acetic acid, TPTZ, ABTS, iron (III) chloride hexahydrate, Trolox, potassium chloride, (+)-catechin, sodium carbonate, F-C reagent, quercetin-3-xyloside, 3,4-dihydroxybenzoic acid, caffeic acid, p-coumaric acid, ferulic acid, sinapic acid, quercetin-3,4′-diglucoside, kaempferol-3-glucoside, catechin-2,3,4-^13^C_3_, ferulic acid-1,2,3-^13^C_3_, glucose, galactose, fructose, raffinose, sucrose, xylose were purchased from Sigma–Aldrich (St. Louis, MO, USA). Quercetin-3-rutinoside, gallic acid, 4-hydroxybenzoic acid, and potassium peroxydisulfate were purchased from Roth (Karlsruhe, Germany). Quercetin-3-glucoside, quercetin-7-glucoside, neochlorogenic acid, cryptochlorogenic acid, kaempferol-3-rutinoside, kaempferol-3-glucoside, kaempferol-7-glucoside were purchased from Extrasynthese (Genay, France). (–)-Epicatechin, chlorogenic acid, rosmarinic acid, quercetin-3-sophoroside, quercetin-3-gentiobioside, quercetin-3-arabinoside, quercetin-3-galactoside, quercetin-4′-glucoside, and quercetin-3-rhamnoside were purchased from Phytolab (Vestenbergsgreuth, Germany). Sodium acetate anhydrous was purchased from CHEMSOLUTE^®^ (Renningen, Germany). Hydrochloric acid from Fisher Chemical (Pittsburgh, UK). Deionized water was from MilliQ apparatus (Bedford, MA, USA). 4-Feruloylquinic acid, 5-feruloylquinic acid, kaempferol-3,4’-diglucoside, kaempferol-3-O-sophoroside were obtained from ChemFaces (Wuhan, China). Sodium hydroxide solution (50% in water) was obtained from Honeywell International Inc. (Charlotte, NC, USA).

### 2.2. Potato Materials

Fresh potato tubers were collected from 14 fields and four commercial retailers. In total, 28 potato samples belonging to 18 different varieties were obtained, and they were harvested in two harvest seasons (2019 and 2020). Two fields were repeated in two different years (2019 and 2020): Oris (BZ) and San Genesio (BZ), respectively. Detailed information is reported in Table 1.

From each sample, three biological replicates, each consisting of three to five tubers (approximately 150 g), were selected and treated separately. The potatoes were cleaned using tap water and dried with tissue paper. Each tuber was cut into small pieces (approximately 4 × 3 cm) without peeling the skin and then, they were steam-cooked.

### 2.3. Steam Cooking Method

Potato samples were cooked using a commercial steamer (Avance Collection Vaporiera HD9150/91, Philips). The cooking method was optimized based on the current literature [14] and the minimum cooking time was used to obtain an adequate palatability and taste in accordance with Italian eating habits [14]. The pieces of each biological replicate were arranged in a circle in the steamer baskets to reach uniform steaming and heating conditions. The food materials were cooked for 20 min. Then, the potato pieces were softly peeled off and stored at −80 ${}^{{}^{\circ}}$C. The samples were freeze-dried, ground to a fine powder in a grinder and stored into air-tight plastic zipper bags at −80 ${}^{{}^{\circ}}$C until the analyses.

### 2.4. Antioxidants, Total Polyphenolic Content, Polyphenols, and Sugars Extraction Method

The extraction protocol was adapted from Tierno et al. [15]. Powdered samples (100 mg) were extracted with 5 mL of methanol-water (70/30 v/v), acidified with HCl (to a final concentration of 0.12 mol/L). The mixture was vigorously shaken for 20 min using a multi-rotator, sonicated for 15 min and again vigorously shaken for 20 min. The supernatant was recovered, and the extraction operation was repeated. The methanolic extracts were combined (10 mL in total), then 1 mL was centrifuged at +5 °C at 14,000 rpm for 15 min. The supernatant was removed and stored at −80 ${}^{{}^{\circ}}$C until the analysis. For each biological replicate, three technical replicates were extracted.

### 2.5. Anthocyanin Extraction Method

The extraction protocol of Oertel et al. [9] was applied. Briefly, 100 mg of freeze-dried material was extracted with a mixture of 500 μL of methanol-water (70/30 v/v acidified with formic acid (0.2 mg/L). The sample was vortexed for 3 s at room temperature, shaken for 30 min on a thermomixer at +4 ${}^{{}^{\circ}}$C and centrifuged for 10 min at +4 ${}^{{}^{\circ}}$C with 12,000 rpm. The supernatant was recovered, and the extraction operation was repeated once again with the pellet. The combined extracts were stored at −80 ${}^{{}^{\circ}}$C until the analysis.

### 2.6. Spectrophotometric Assays

#### 2.6.1. Total Polyphenolic Content (TPC)

The TPC was determined by the Folin–Ciocalteu method and adapted from Valls et al. [16]. 250 μL of deionized water and 60 μL of extracts was added to 60 μL of Folin–Ciocalteu reagent. The mixture was mixed at 12,000 rpm for 6 min at room temperature (RT). Then, 630 μL of sodium carbonate (7.5% *w*/v) were added to the mixtures, and they were mixed at RT for 90 min. The absorbance was recorded at 740 nm on a Cary 60 UV–Vis (Agilent Technologies, Palo Alto, CA, USA) spectrophotometer, and referred to a standard curve of gallic acid (range 0–150 ng/μL). The results were normalized for the weight and expressed as mg of gallic acid equivalents per 100 g of fresh weight sample (Table S3).

#### 2.6.2. Antioxidant Activity, ABTS Assay

It was determined using Trolox equivalent antioxidant capacity (TEAC) assay adapted from Valls et al. [16]. For the assay, 1970 μL of ABTS reagent was added to 30 μL of sample extract and the mixture was incubated into dark for 10 min at RT. The decrease in absorbance was read at 734 nm using a Cary 60 UV–Vis (Agilent Technologies, Palo Alto, CA, USA) spectrophotometer, and referred to a standard curve of Trolox (range 15.6–250 ng/μL). The results were normalized for the weight and expressed as mg of Trolox equivalents per 100 g of fresh weight sample (Table S3).

#### 2.6.3. Antioxidant Activity, FRAP Assay

It was determined by FRAP assay adapted from Valls et al. [16]. The purple-fleshed samples were prepared for the assay by diluting them 1:10 using the extraction solution (Section 2.4), whereas, the extracts of the yellow-, white-fleshed, and red-skinned potatoes were used without any further dilution. For the assay, 960 μL of FRAP reagent was added to 60 μL of sample extract and 180 μL of MilliQ. The mixtures were incubated into dark at +37 ${}^{{}^{\circ}}$C for 90 min. The decrease in absorbance was read at 595 nm using a Cary 60 UV–Vis (Agilent Technologies, Palo Alto, CA, USA) spectrophotometer, and referred to a standard curve of Trolox (range 15.6–250.0 ng/μL). The results were normalized for the weight and expressed as mg of Trolox equivalents per 100 g of fresh weight sample (Table S3).

#### 2.6.4. Total Monomeric Content and Total Anthocyanins Content (Anto Mono and Anto Tot)

The Anto mono and Anto tot were determined by pH differential method and adapted from Valls et al. [16]. Two dilutions of the same extract were made by adding 800 μL of potassium chloride (0.025 M, pH 1) to 200 μL of sample extract and 800 μL of sodium acetate (0.4 M, pH 4.5) to 200 μL of sample extract, respectively. The absorbance was immediately recorded at 520 and 700 nm on a Cary 60 UV–Vis (Agilent Technologies, Palo Alto, USA) spectrophotometer. The calculations were conducted using Lambert–Beer law (ε = 26,900 L/mol/cm, MW = 449.2 g/mol). The results were normalized for the weight and expressed as mg of cyanidin-3-glucoside equivalents per 100 g of fresh weight sample (Table S4).

### 2.7. Analysis of Polyphenolic Compounds

The analytical procedure was adapted from Ieri et al. [17]. An UltiMate 3000 UHPLC system (Thermo Scientific, Waltham, MA, USA) coupled with a TSQ Quantiva (Thermo Scientific, Waltham, MA, USA) triple-stage quadrupole mass spectrometer was used (UHPLC-QqQ-MS/MS). The quality control sample (QC) was made by pooling 500 μL of one yellow-fleshed variety extract, 500 μL of one white-fleshed variety extract, 500 μL of one red-skinned variety extract, and 500 μL of one purple-fleshed variety extract. Then, the resulting mixture was diluted 1:10 with extraction solution and used to control the absence of chromatographic drift and to check the stability of the instruments. A blank sample (solvent mixture) was used to verify absence of significant carry-over effect. The procedure for the calibration curve is adopted by Vrhovsek et al. [18]. The mother solutions of each analytical standard (2000 ng/μL) were prepared using methanol or a mixture of methanol/water (50/50 v/v. Four starting standard mixtures were prepared at 50 ng/μL representing the most known polyphenolic compounds (Table S1) following the current literature [2,18]. These starting standard mixtures were serially diluted to working concentrations. The calibration curve of polyphenolic standards covered a range between 10.0 and 0.0025 ng/μL. A mixture of two internal standards, which was prepared at 2 ng/μL using catechin-2,3,4-^13^C_3_ and ferulic acid-1,2,3-^13^C_3_, was used to monitor the instrument performance and an overall RSD% of 4.34% and RSD% 6.84% was respectively found. All potato samples were diluted 1:10 with extraction solution before analysis. Each injected solution was prepared by mixing 50 μL of potato sample (or QC, or standard mixture) with 50 μL (2 ng/μL) of internal standard solution, and they were analyzed in a randomized order, each 10 analyzes a QC was injected. Repeatability (intra-day and inter-day precision) was measured as RDS% after multiple injections (*n* = 10) of mixture of the standards at 1 ng/μL, 0.1 ng/μL, and 0.01 ng/μL. The RSD% for all the standards was calculated, which have been injected 10 times inter-day [17]. The mobile phase was B (0.1% [v/v] formic acid in acetonitrile LC-MS analytical grade) and A (0.1% [v/v] formic acid in Millipore water). The gradient elution was 0–1.5 min (5% B), 1.5–8 min (25% B), 8–10 min (55% B), 10–11 min (95% B), 11–12 min (95% B), 12–12.1 min (5%) with a flow rate 0.3 mL/min. The autosampler and column temperature were set at +5 ${}^{{}^{\circ}}$C and +40 ${}^{{}^{\circ}}$C, respectively. The separation of the compounds was performed using a Hypersil GOLD™ HPLC (2.1 × 100 mm, 1.9 μm, Thermo Scientific, Waltham, MA, USA) column with the corresponding pre-column. Injection volume was 1.5 μL. The operations were controlled by the Chromeleon Chromatography Data System (CDS) (version 6.8) software and Thermo Xcalibur (version 3.0) software (both Thermo Scientific, Waltham, MA, USA). The electrospray ion (ESI) source conditions were as follows: positive voltage 4000 V, negative voltage 3500 V, vaporizer temperature 300 ${}^{{}^{\circ}}$C, capillary temperature 325 ${}^{{}^{\circ}}$C, sheath gas 40 arbitrary unit (AU), auxiliary gas 10 AU, sweep gas 0 AU. The collision gas (Argon) was set at 1.5 mTorr. Data processing was conducted using Tracefinder 3.2. The method was able to detect 27 polyphenolic compounds and a total of 15 polyphenols was quantified in the potato varieties under investigation. The retention time, ESI polarity, molecular weight, precursors, product ions, collision energy, RF lens, regression parameters, and linearity range of the calibration curve of each compound was summarized in Table S1. The quantification of each analyte was carried out by an external calibration curve using analyte/internal standard area ratios. The results were normalized for the sample weight and expressed as mg of each compound per 100 g of fresh weight sample (Table S3).

### 2.8. Analysis of Sugar Compounds

The analytical procedure was adapted from Eisenstecken et al. [19]. An ion chromatograph (IC) with pulsed amperometric detection (HPAE-PAD) was used to quantify individual sugars. The analysis was performed on ICS-5000 (Thermo Scientific Dionex, Sunnyvale, CA, USA). The separation of sugars was achieved by a Dionex CarboPac PA1 Analytical column (4 × 250 mm) and a Dionex CarboPac PA1 Guard column (4 × 50 mm). Yellow- and white-fleshed potato extracts were diluted using the extraction solution (Section 2.4) 1:10 before the injection, whereas, the extracts of the red-skinned and purple-fleshed potato samples were used without any further dilution. The calibration curve of sugar standards covered a range between 200.0 and 0.1 ng/μL. An isocratic condition with 50 mM sodium hydroxide (solvent A, 80%) and Millipore water (solvent B, 20%) for 17 min was used for the chromatographic separation of the sugar compounds, followed by a regeneration of the column with 200 mM NaOH (solvent C, 100%) for 10 min. Flow rate was set at 1.2 mL/min, injection volume was 20 μL and the column temperature +30 ${}^{{}^{\circ}}$C. An Au on PTFE disposable working electrode and a pH-Ag/AgCl reference electrode were used. All compounds were quantified with the calibration curve of each sugar. The operations were controlled by the Chromeleon Chromatography Data System (CDS) (version 6.8, Thermo Scientific, Waltham, MA, USA) software. The analytical features could be found in Table S5. The results were normalized for the sample weight and expressed as mg of each compound per 100 g of fresh weight sample (Table S3).

### 2.9. Analysis of Anthocyanins

The analytical procedure was adapted from Oertel et al. [9]. The analysis was performed on red-skinned and purple-fleshed potato samples. Separation was performed on a Waters Acquity UPLC system (Milford, MA, USA) using a RP Acquity UPLC BEH C18 column (130 A°, 2.1 × 150 mm, 1.7 μm, Waters, Milford, MA, USA), protected with an Acquity UPLC BEH C18 pre-column (130 A°, 2.1 × 5 mm, 1.7 μm, Waters, Milford, MA, USA). The following multistep linear gradient was used: 0–4 min (5–40% B), 4–9 min (40–55% B), 9–11 min (55–95% B), and an isocratic hold for 3 min. Solvent B was methanol/5% formic acid [v/v] and solvent A was water/5% formic acid [v/v], administered at a flow rate of 0.3 mL/min. The injection volume was 2 μL for both sample and standard solutions. Samples were kept at +6 ${}^{{}^{\circ}}$C during the analysis, the column at +40 ${}^{{}^{\circ}}$C. Mass spectrometry detection was performed on a Waters Xevo TQMS (Milford, MA, USA) instrument equipped with an electrospray (ESI) source. Capillary voltage was 3.5 kV in positive mode, the source was kept at 150 ${}^{{}^{\circ}}$C; desolvation temperature was 500 ${}^{{}^{\circ}}$C, cone gas flow, 50 L/h, and desolvation gas flow, 1000 L/h. Unit resolution was applied to each quadrupole. MRM (multiple-reaction monitoring) data analysis was conducted using MassLynx 4.1. All MRM parameters parameters and acronyms can be found in Table S2. All compounds were quantified with the calibration curve of cyanidin 3-glucoside. The results were normalized for the sample weight and expressed as mg cyanidin 3-glucoside per 100 g of fresh weight sample (Table S4).

### 2.10. Datasets

To conduct the data analysis in this study, the following datasets were generated: antioxidants (AA), polyphenols (poly), and sugars (sug) (Table 2).

### 2.11. JIVE, a Data Fusion Approach

Statistical analysis was performed using R software version 4.1.1. (R Foundation for Statistical Computing, Vienna, Austria) [20]. The shared and unshared components across the three datasets were investigated using the unsupervised multivariate approach Joint and Individual Variation Explained (JIVE) [12]. The missing values were imputed in all three datasets AA, poly, and sug (Table 2) by replacing them with a random value between zero value and the LOD (limit of detection) of the analytical method. The data were log-transformed, mean-centered, and scaled before JIVE analysis. The function used is called jive of the JIVE package [12]. The selection of the number of components was done by a permutation test to identify the number of components for common and distinct variation. The number of components were known as “ranks” [12], which were 1, 1, 3, 1 for joint, AA, poly, and sug datasets, respectively. Then, the JIVE results were submitted to the PCA (Principal Component Analysis) function of FactomineR package [21,22] to calculate loadings and score values. Based on the calculated rank, the loadings were extracted by the PCA results, and they were summarized in one dimension by using the facto_summarize function in R [22], which is able to subset and summarize the results of PCA. The resulting loadings from the facto_summarize function were used to calculate the variable importance of each polyphenol. The variable importance of each metabolite is calculated based on the results obtained by the variable’s coordinates on the ranks and the dimension’s eigenvalue’s on the PCA results [22].

## 3. Results and Discussion

### 3.1. Interpretation of JIVE Model

**Figure 1:**
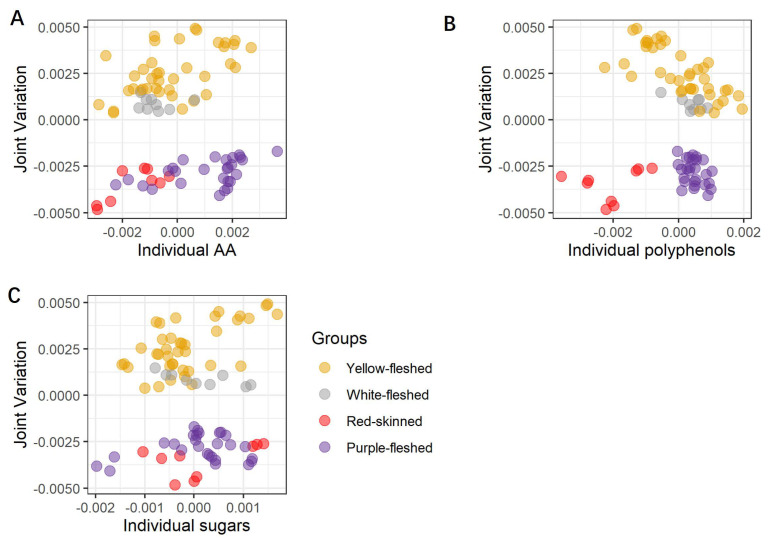
Score plots of the joint part of the three joined datasets, and the individual parts of the three separated datasets are reported as joint and poly (**A**), joint and sug (**B**), and joint and AA (**C**). The joint part gathers all three datasets (poly, sug, AA). The poly dataset includes the individual polyphenols, the sug dataset includes the individual sugars, and the AA dataset includes ABTS, FRAP, and FC assays. The 28 potato samples are organized into four groups (yellow-, white-fleshed, red-skinned, and purple-fleshed), which are highlighted using four different colors yellow, grey, red, and purple, respectively. For each steam-cooked potato sample, the three corresponding biological replicates are graphically presented as points and using the corresponding group color. AA = antioxidant activity, Poly = single polyphenols, Sug = sugars.

JIVE is essentially a latent variable (LV) model, so its results can be graphically represented by score plots (similar to the one obtained by principal component analysis (PCA)) [12], in which the points represent the individual potato samples. The score plots show the projection of the data onto a set of “principal” components which represent joint and individual LVs. The joint component captures the variability common to the jointed datasets, while the individual components highlight the unique contributions of each dataset. In the score plots, the joint component is represented in the *y*-axis, whereas the *x*-axis is changed for each individual component of each dataset. These combined plots are used to investigate the similarity and/or dissimilarity amongst datasets. These score plots are shown in Figure 1 and this representation is known as a one-versus-the-rest approach.

**Figure 2:**
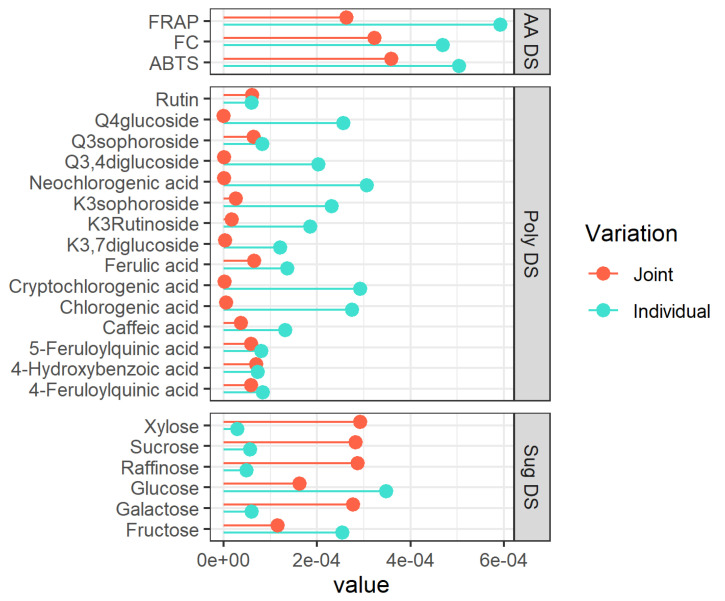
Variable importance (VIP) for each metabolite of the individual (turquoise lines) and Joint (tomato lines) parts. The VIP is calculated based on the rank extracted from the JIVE results. The ranks are 1, 3, 1 for the three separated datasets (AA, poly, and sug), respectively, whereas the joint part has a rank equal to 1 (see Section 2.11). AA = antioxidant activity, Poly = single polyphenols, Sug = sugars, DS = dataset, K = Kaempferol, Q = Quercetin.

Looking at Figure 1A–C, a clear separation between the non-pigmented and pigmented steam-cooked potato samples is recognizable along the joint component, thus indicating that this type of partitioning is common to all three datasets. Further separation between the red-skinned and purple-fleshed samples is also visible (Figure 1B), but this separation is now concentrated along the individual components of the polyphenolic dataset, thus demonstrating that this class of compounds is exclusively able to differentiate among the two pigmented groups. Interestingly, the combination of polyphenols and common variation seem also to clearly separate the individual red-skinned varieties (local red variety, Desiree, and Red Scarlett), but a similar level of partitioning is not visible for the other three macro groups, thus providing evidence that each red-skinned potato may have a characteristic and distinctive metabolome. Looking at the individual component of the sugar dataset, a peculiar trend is observed for the red-skinned local red variety and the purple-fleshed Vitelotte, whose behavior prevents a complete separation of red-skinned and purple-fleshed varieties similar to the one observed for the polyphenols. The plot suggests that in terms of sugars, these two varieties are significantly divergent in contrast to the other members of the corresponding groups. The inspection of the variable importance in the different components of the JIVE model can be used to assess the specific contribution of the different metabolites and classes of metabolites to the individual and common components of the variability. In our case, the variable importance (VIP) of each compound is calculated using the reference [22] and shown in Figure 2.

The plot (Figure 2) suggests that the trends in the polyphenols are mainly captured by the individual component of the model (10 out of the 15 compounds showed a higher VIP for this component), while the variability of sugars mainly goes in the common part. In terms of interpretation, this means that the separation between pigmented and non-pigmented potatoes should be clearly visible in the sugar dataset, while the separation within the two pigmented groups should show up in the polyphenols dataset. Regarding the sugar dataset (sug DS), high values of VIPs for xylose, sucrose, and raffinose are observed demonstrating their responsibility in the separation between pigmented and non-pigmentated samples in the joint structure. On the contrary, considering the polyphenolic dataset (poly DS), high values of VIPs for the neochlorogenic, cryptochlorogenic, and chlorogenic acids are found, thus suggesting that they carry considerable value in the separation between pigmented and non-pigmentated samples too. In the case of the antioxidants, the picture is less clear-cut since this layer of data seemed to contribute almost in the same way to the individual and common components. A detailed discussion of the trends of the individual metabolites will be the subject of the following section.

### 3.2. Individual Metabolites

#### 3.2.1. Antioxidant Activities and Total Polyphenolic Content

**Figure 3:**
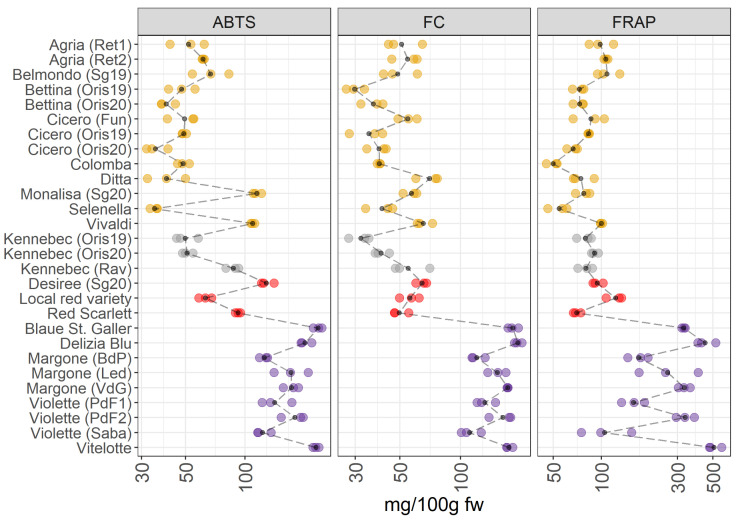
ABTS, FC, and FRAP in 28 potato varieties organized in four groups (yellow-, white-fleshed, red-skinned, and purple-fleshed); three biological replicates for each sample were analyzed. The black points represented the averages for each analyte calculated on three biological replicates. The black long dash lines showed the concentration trends amongst all the potato groups. The concentrations were transformed in a log-10-scale. fw = fresh weight. For more detailed information about potato varieties see Table 1.

Figure 3 shows the antioxidant capacity (ABTS and FRAP assays) and the total polyphenols content (Folin–Ciocalteu (FC) assay) across the four groups of potatoes.

The VIPs of the JIVE model (Figure 2) indicate that ABTS, FC and FRAP are mainly contributing to the individual part of the model, so a clear separation between between pigmented and non-pigmented varieties should not be the clearest structure in this dataset. This is confirmed by Figure 3, which shows a comparable amount of antioxidants and in the total polyphenolic content between yellow-, white-fleshed, and red-skinned groups. In contrast, the purple-fleshed potatoes have the highest antioxidant contents, in particular in the case of FC. Looking at the individual varieties, Vivaldi and Monalisa collected from San Genesio (Sg20, BZ, Italy) in 2020 (as yellow-fleshed potatoes), demonstrate higher values in the antioxidant capacity assay (ABTS), than the others yellow-fleshed samples. Additionally, the white-fleshed potatoes show a comparable content of antioxidants to yellow-fleshed samples, however, Kennebec from Rav (Ravina, TN, Italy) shows higher values of antioxidant activities (ABTS assay) than the other Kennebek cultivars collected in Oris (BZ, Italy). Additionally, there is a marked variability in the concentration of antioxidants in the red-skinned and purple-fleshed groups. Vitelotte and Blaue St. Galler cultivars, both belonging to the purple-fleshed group, show the highest levels of antioxidants and total polyphenolic content.

Years: Cicero, Bettina, and Kennebec potato varieties, which were cultivated in the same environment conditions (Oris, BZ, Italy), have been harvested in two consecutive years (2019 and 2020). These samples are indicated in the Figure 3 as Oris19 and Oris20 that means Oris-grown potatoes in 2019 and 2020. Differences in the total polyphenolic content and antioxidant properties were hardly observed both in yellow-fleshed (Cicero, Bettina) and in white-fleshed (Kennebec) potatoes.

Environmental conditions: In agreement with the published literature, our data show a noticeable variability in intra- and inter-groups (Figure 3). The intra-group variability is linked to environmental conditions which influences the accumulation of polyphenols in the crop [23]. At the same time, Lachman et al. [24] reported that there were significant differences in total polyphenolic content depending on the different places of growth.

The accumulation of phenolic compounds is regulated by the potato genotype, and several types of phenolics are synthesized. Additionally, the antioxidant potential of phenolic metabolites is influenced by the position of hydroxylation [23] and by the mean relative concentrations of the antioxidants found in the vegetable matrices. Several authors reported that the purple-fleshed varieties were characterized by a high content of antioxidants, and that the content in the pigmented varieties may be 2-3 fold higher than in non-pigmented potatoes [5,25]. This was linked to the high content of polyphenols and anthocyanins, which demonstrated to have strong antioxidant properties [7,25,26]. Deuber et al. [27] reported that Vitelotte cultivar was characterized by the highest polyphenol and antioxidant content, thus confirming our results [23,27]. Furthermore, the same pattern is reported previously by Hamouz et al. [28], where they analyzed Vitelotte, Violette, and Blaue St. Galler varieties showing a very high content of antioxidants. Contrary to what is stated by Hamouz et al. [28], the Violette variety from Sabaudia (Saba) (LT, Italy) shows the lowest polyphenol content and antioxidants in the purple-fleshed group considered in this survey. The antioxidant activity was lower in the red-skinned potatoes due to the lower content in anthocyanins compared to purple-fleshed varieties [28]. The health effects of antioxidant compounds, mediated by their radical scavenging activity, are well-known [29] and other studies reported that the pigmented potato varieties had the highest antioxidant capacity [9,28,30]. Thus suggesting that these cultivars for selection for a healthy human diet.

#### 3.2.2. Polyphenolic Compounds

**Figure 4:**
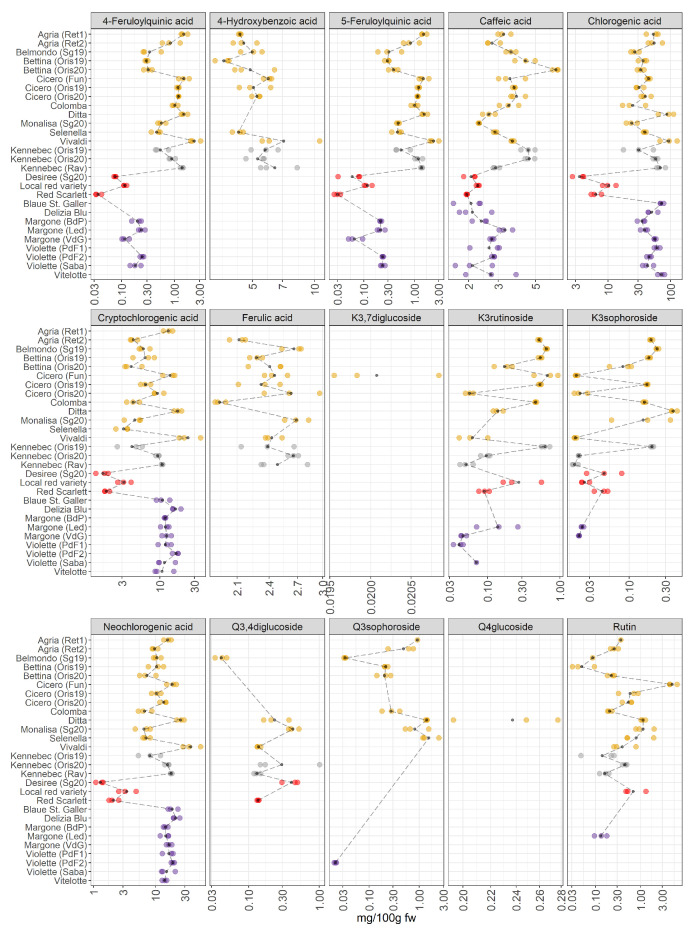
Polyphenolic compounds in 28 potato varieties organized in four groups (yellow-, white-, red-skinned, and purple-fleshed); three biological replicates for each sample were analyzed. The black points represented the averages for each analyte calculated on three biological replicates. The black long dash lines showed the concentration trends amongst all the potato groups. The concentrations were transformed in a log-10-scale. K = Kaempferol, Q = Quercetin, fw = fresh weight. For more detailed information about potato varieties see Table 1.

An interesting variability of polyphenolic compounds is found amongst the potato groups under investigation (Figure 4).

JIVE has indicated a strong role of this dataset in the differentiation of red-skinned and purple-fleshed potatoes (Figure 1B), and many polyphenols show a high importance in the individual component of the model. This differentiation should be then visible at the level of raw concentrations. Individual polyphenols (Figure 4) indeed show that red-skinned potatoes have the lowest concentration of many phenolic compounds. As already discussed, red-skinned varieties show very characteristic profiles.

As far as the individual metabolites are concerned, the plot shows an interesting variation in the concentration of 4-feruloylquinic and 5-feruloylquinic acids, which have been hardly considered in the literature [8] due to the scarce availability of pure certificated analytical standards. These metabolites are quantified and detected in a few pigmented samples: Violette variety from Piana del Fucino2 (PdF2) (AG, Italy) and Sabaudia (Saba) (LT, Italy) and in the variety of Margone collected from three cultivation areas in Trentino (Baselga di Pinè (BdP), Val di Gresta (VdG), and Val di Ledro (Led) (TN, Italy)), showing an appreciable concentration of 4-feruloylquinic and 5-feruloylquinic acids; in contrast, these two phenolic compounds are not detected in the cultivar Blaue St. Galler, collected in Val Pusteria (BZ, Italy). It is important to highlight that the individuals VIP of 4-feruloylquinic and 5-feruloylquinic acids (Figure 2) were slightly higher than the VIP of the joint component because these compounds were not detected in some cultivars (the missing values are imputed before the JIVE analysis). The reasons for the lack of quantification of some metabolites (4-hydroxybenzoic acid, 4-feruloylquinic acid, 5-feruloylquinic acid, ferulic acid, kaempferol and quercetin derivatives) in some potato cultivars was because these metabolites were present only in traces or were absent. Thus, meaning that the concentration of a specific compound was generally lower than its limit of quantification (LOQ). The LOQ of each analyte is reported in Table S1. Within the yellow-fleshed samples, considerable variability in the concentration of hydroxycinnamic acids (chlorogenic, cryptochlorogenic, and neochlorogenic acids) was found. The variety Vivaldi significantly stands out among the yellow-fleshed varieties due to the highest concentration of all isomers of chlorogenic acid. Additionally, this cultivar shows high antioxidant activity (Figure 3), and the positive correlation between the chlorogenic acids and antioxidant activities has been already reported [31]. Within the red-skinned group, the local red variety shows the highest amount of hydroxycinnamates acids. The amount of these hydroxycinnamic acids is comparable to the concentrations found in white-fleshed samples. It is recognized that there is a noteworthy amount of hydroxycinnamic acids in the purple-fleshed samples and the levels of these metabolites are comparable to the concentrations in yellow- and white-fleshed potatoes. Surprisingly, the concentration of hydroxycinnamic acids is strongly different between red-skinned and purple-fleshed samples. Therefore, our data confirm that the tissue-specific accumulation of some hydroxycinnamic acids may be cultivar-specific as well [8,9]. The VIP of hydroxycinnamic acids turned out to be extremely higher in the individual component than the joint ones, thus suggesting that they are the most influential factors in separating red-skinned and purple-fleshed groups (Figure 2). No missing values are reported (Figure 4). Some of the polyphenols strongly accumulate in yellow- and white-fleshed potatoes, especially kaempferol and quercetin derivatives. Indeed, our data show a pronounced variability in the concentration of kaempferol glycosides, and they are mostly present within yellow- and white-fleshed groups. Kaempferol-3-rutinoside and kaempferol-3-sophoroside are detected in all red-skinned varieties and a few purple-fleshed samples: Violette cultivar from Sabaudia (Saba) (LT, Italy) and Piana del Fucino1 (PdF1) (TN, Italy), and Margone cultivar (VdG and Led (both TN, Italy)). The VIP of kaempferol-3-rutinoside and kaempferol-3-sophoroside (Figure 2) are higher in the individual part, thus demonstrating that the presence of these two kaempferol glycosides may be used to distinguish the two pigmented groups considered for this survey. Regarding the quercetin glycosides, they are mostly detected in the yellow- and white-fleshed potato groups. Rutin, the most abundant quercetin found in potatoes [7,32], is also quantified in the local red variety (TN, Italy) and in the Violette from Piana del Fucino2 (PdF2) (TN, Italy). The quercetin-3,4-diglucoside is found in all red-skinned varieties and its amount is comparable with the concentration found in yellow- and white-fleshed samples. The VIP of rutin in the individual and joint parts are comparable, indeed, a modest variability amongst the yellow-, white-fleshed and red-skinned groups is observed (Figure 2). In contrast, the individual VIP of quercetin-3,4-diglucoside is extremely higher than the joint VIP because the missing values are mostly imputed.

Years: Regarding the three varieties Cicero, Bettina, and Kennebec collected from Oris (BZ, Italy) in 2019 and 2020, the sampling year hardly influenced the concentration of polyphenols. Little variability in the concentration of hydroxycinnamic acid is observed. On the contrary, the amount of kaempferol-3-rutinoside and kaempferol-3-sophoroside is affected by the harvest year in the three cultivars under investigation. Indeed, the concentration of these compounds is higher in those cultivars collected in 2019 than in those harvested in 2020. In contrast, the rutin seems to be less variable between the two years. As confirmed by Reddivari et al. [33], the influence of the genotype on the polyphenolic profile was more significant than the effects given by specific climatic features of the given year. Nevertheless, it is worth reporting that the amount of polyphenols varied across their potato samples [33], thus supporting our results that show the effect of two years on the polyphenolic compounds concentrations in Cicero, Bettina, and Kennebec cultivars (Figure 4).

Environmental conditions: It is worth noting that our data show a remarkable variability in the concentration of phenolic compounds in potato samples belonging to the same varieties (Kennebec, Agria, Margone, and Violette), which are collected from different growing areas. Payyavula et al. [34] reported that the concentration of phenolics varied among the locations, and our data were in agreement with the authors [34,35]. Remarkably, the cultivars Kennebec, Cicero, and Bettina, which are harvested from Oris (BZ, Italy), show nearly the same concentration of polyphenols.

Chlorogenic acid is the major compound detected and quantified among the polyphenolic compound classes in all potato samples under investigation, and our findings are in accordance with the literature [14,32,34]. This bioactive compound had a strong antioxidant activity, indeed, the purple-fleshed potatoes have shown the highest amount in antioxidants (Figure 3). Deuber et al. [27] found the highest chlorogenic acid content in the Vitelotte cultivar in agreement with our study [27]. Additionally, Blaue St. Galler demonstrated a relevant concentration of chlorogenic acid (Figure 4). As reported by Ingallina et al. [8], chlorogenic acid was the strongest antioxidant compound, and the highest content of this metabolite was found in our study in Ditta, Vivaldi, and Vitelotte cultivars. Bassoli et al. [36] demonstrated that chlorogenic acid was able to inhibit glucose-6-phosphatase, which is involved in the pathway of gluconeogenesis and glycogenolysis and in the release of glucose in the blood controlling glycemia in type 2 diabetics. Therefore, the identification of those cultivars, in which the concentration of chlorogenic acid is high, could be potentially powerful for diabetic people. In the last decades, the attention towards phytochemicals has increased due to their well-known healthy properties [2]. Several authors reported the antioxidant properties of polyphenols, and their healthy properties have been deeper examined [2]. The antioxidant, anticancer, antiproliferative, and anti-inflammatory effects of potato extracts were studied in vivo and in vitro [7]. The two most abundant flavonols were rutin and kaempferol-3-rutinoside as reported by the review of Akyol et al. [2]. Surprisingly, Rommens et al. [13] tried to increase the production of kaempferol-3-rutinoside by modulating the flavonoid biosynthetic pathway. These metabolites undergo different metabolic transformations after the intake of flavonoid-rich foods [37]. A high amount of flavonoids is currently associated with different healthy properties. For example, the presence of quercetin derivatives may be useful for cardiovascular disease prevention and kaempferol derivatives are characterized by anti-inflammatory activities [37].

#### 3.2.3. Sugars and Polyols

**Figure 5:**
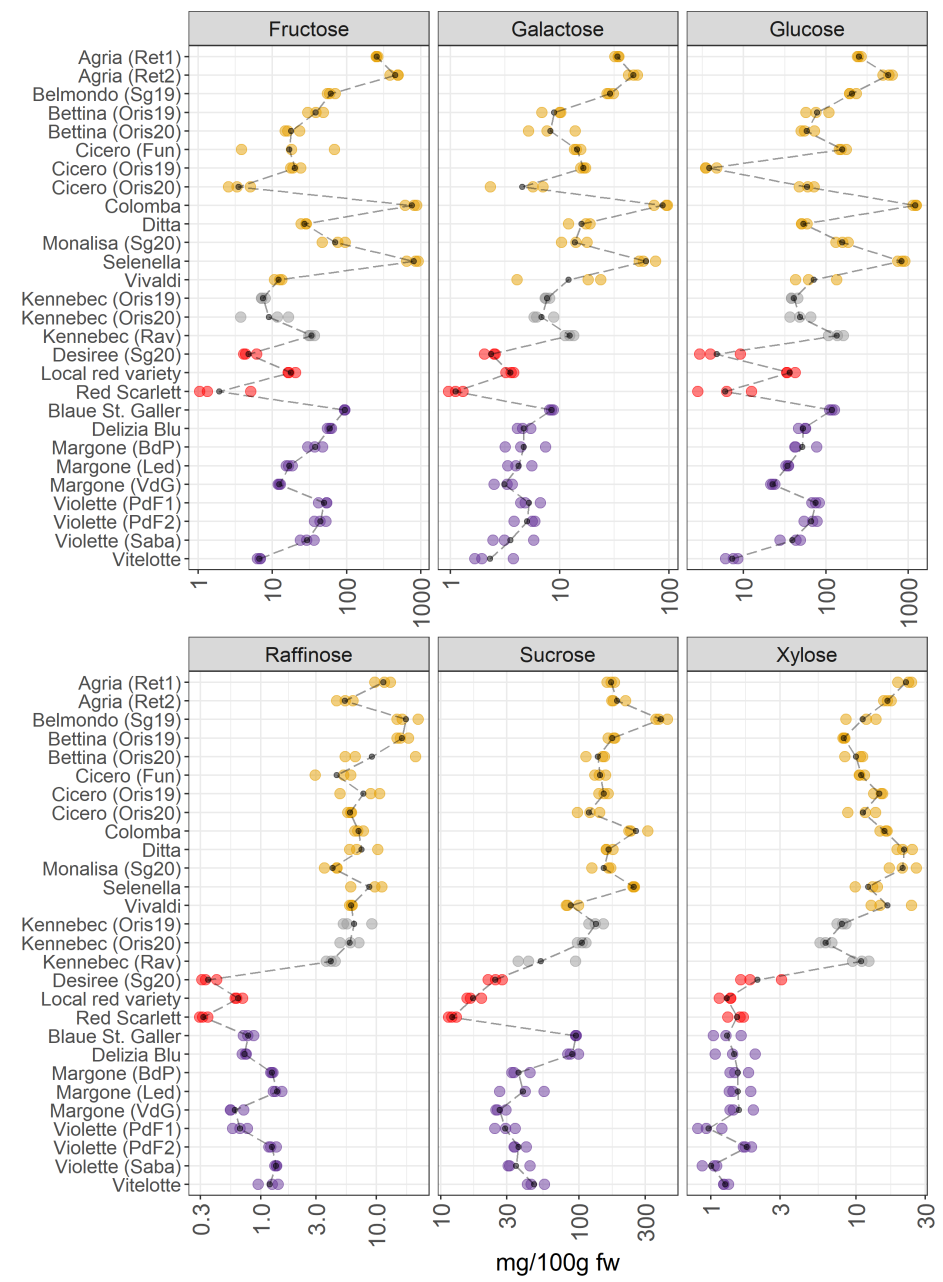
Content of individual sugars in 28 potato samples organized in four groups (yellow-, white-, red-skinned, and purple-fleshed); three biological replicates for each sample were analyzed. The black points represented the averages for each analyte calculated on three biological replicates. The black long dash lines showed the concentration trends amongst all the potato groups. The concentrations were transformed in a log-10-scale. fw = fresh weight. For more detailed information about potato varieties see Table 1.

The main individual sugars quantified in potato samples were fructose, galactose, glucose, raffinose, sucrose, and xylose and are reported in Figure 5.

The VIPs of JIVE (Figure 2) indicated that some of the members of this metabolic class (raffinose, xylose, sucrose, and galactose) were almost exclusively contributing to the joint part of the model. This result clearly shows up in the individual trend, where pigmented varieties are characterized by a strong reduction of the sugars raffinose, xylose, and sucrose (Figure 5). The profiles of glucose, galactose, and fructose showed a notable variability within the yellow-fleshed potato group. Conversely, the other three sugars show a lower variability within the same macro group. The white-fleshed potato samples show a comparable concentration of these carbohydrates with the yellow-fleshed group. As already pointed out during the discussion of the JIVE results, the trends observed for the red local variety and Vitelotte are somehow extreme in comparison to the other members of the corresponding groups, thus providing evidence that the metabolome of these potato samples is peculiar and distinctive. In detail, the amount of sugars in the local red variety is comparable to the concentration of the carbohydrates found in the purple-fleshed varieties and this sample has the highest amount of glucose and fructose within the red-skinned group (Figure 5). Indeed, the scores show an interesting behavior of this potato sample in the individual component of the sugar dataset (Figure 1C), which is separated by the other red-skinned samples. Whereas the amount of sugars of Red Scarlet and Desiree varieties is the lowest detected amongst the groups. Another interesting observation may be done evaluating the trend observed for the Vitelotte cultivar, as a purple-fleshed variety, that demonstrates an interesting variation in the individual sugar profile. The amount of glucose and fructose is the lowest found within the purple-fleshed group and the Vitelotte sample has a sugar profile comparable to the red-skinned varieties. The scores of the Vitelotte cultivar are closer to the red-skinned group and it is well-differentiated by the other purple-fleshed samples (Figure 1C). The VIP of glucose and fructose are more influential in the individual component (Figure 2), thus suggesting that the concentration of these two carbohydrates may be useful to distinguish the red-skinned and purple-fleshed groups considered in this survey. Looking at the panels of the concentration of raffinose, sucrose, and xylose (Figure 5), the separation between non-pigmented and pigmented samples is clearly visible. These findings are confirmed by both what is observed in the score plot (Figure 1C), in which the two macro-groups are sharply noticeable, and the VIP that mainly has influential importance in the joint component (Figure 2).

Years: For Cicero, and Bettina, yellow-fleshed varieties obtained from Oris (BZ, Italy) in 2019 and 2020, a pronounced variation in the levels of sugars is observed. In contrast, the two Kennebec samples collected in Oris in 2019 and 2020 do not show differences in the levels of sugars. The reason could be linked to the different meteorological conditions and these cultivars react differently to the seasonal variation.

Environmental conditions: Looking at the same varieties of potatoes (Kennebec, Agria, Violette, and Margone), which are collected from different places, a slight variation is observed amongst these varieties within the groups. A more pronounced variation in the concentration levels of sugars is noticed in Kennebec and Margone samples. In detail, the Kennebec variety collected from Rav (TN, Italy) showed higher levels of sugars than those collected in Oris (BZ, Italy). It is worth noticing that the difference of altitude of the corresponding growing areas is about 600 m between Rav (around 200 m a. s. l.) and Oris (875 m a. s. l.). Similar results were observed in the study conducted by Andre et al. [35], where the influence of climatic cultivating conditions and altitude effects were investigated across different potato cultivars. Additionally, the most interesting differences in the glucose content between the two Agria samples obtained from commercial retailers are found.

It is well-known that fructose and glucose are produced in potatoes in equimolar amounts, even though the concentration of glucose is slightly higher than fructose (Figure 5). Glucose and fructose are reducing sugars and sucrose is a non-reducing sugar [38]. It is well known that the levels of these sugars depended on several factors (soil moisture, genotype, the levels of precipitation, irrigation, mineral nutrition, harvest time, and storage conditions) [38] and they are determining the quality of potatoes after storage and cooking and/or industrial processing [38]. Sowokinos et al. [38] reported that the sucrose content in different varieties was variable, and it depended on the reducing sugar (glucose and fructose) accumulation. Additionally, during storage, the carbohydrates were converted from starch, and the major sugars (glucose, fructose, and sucrose) are accumulated [39]. Therefore, the levels of sucrose provided important information in terms of harvest time, maturity, utilization practices, and the quality of potatoes for the selection of more suitable varieties for consumer preferences. Additionally, the potato cultivars containing less sugar should be preferred from consumers to reduce the impact on blood sugar. Therefore, the selection of potato varieties, which contain the lowest content of carbohydrates, could play a potential role in preventing the onset of several dangerous health problems, including obesity and type 2 diabetes.

#### 3.2.4. Anthocyanins

**Figure 6:**
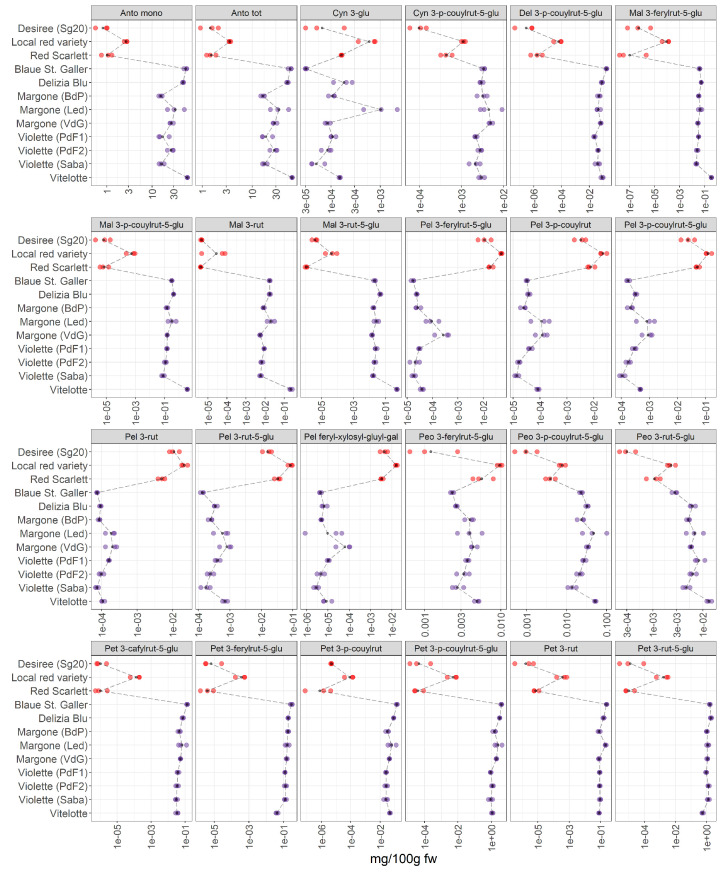
Results of pH differential spectrophotometric method (Anto mono and Anto tot) and 22 single anthocyanins in 12 potato pigmented samples organized in two groups (red-skinned and purple-fleshed); three biological replicates for each sample were analyzed. The black points represented the averages for each analyte calculated on three biological replicates. The black long dash lines showed the concentration trends amongst all the potato groups. The concentrations were transformed in a log-10-scale. fw = fresh weight. Acronyms were reported in Table S2. For detailed information about potato varieties see Table 1.

This last section focuses on a detailed investigation of the accumulation of anthocyanins in pigmented potatoes. Our results complement the studies already performed in recent years [5,7,9]. For this purpose, three red-skinned (Desiree, local red variety, and Red Scarlett) and five purple-fleshed varieties (Blaue St. Galler, Delizia Blue, Margone (from BdP, Led, and VdG), Violette (from PdF1, PdF2, and Saba), and Vitelotte) are collected, in total 12 samples of pigmented potatoes are thoroughly investigated in this study. Detailed information is reported in Table 1. The results are summarized in Figure 6.

We are able to detect and identify 22 anthocyanins in the potato extracts (Figure 6). The list of all acronyms can be found in Table S2. Our data show a pronounced difference between the accumulation of anthocyanins in red-skinned and purple-fleshed potatoes. Inter-varietal differences are observed, and a more pronounced variation in the content of anthocyanins is noted within the red-skinned potato varieties, showing that the local red variety have the highest levels of pigments in this group.

*Total anthocyanin content and total monomeric anthocyanins*: the total anthocyanin content (Anto tot) and total monomeric anthocyanins (Anto mono) in potatoes in the present study were analyzed by the pH differential spectrophotometric method. Generally, the highest content in monomeric anthocyanins and in total anthocyanins is found in purple-fleshed potato varieties. The Vitelotte, Blaue St. Galler and Delizia Blu cultivars turned out to be the varieties with the highest content of total anthocyanins and total monomeric anthocyanins. Additionally, within the red-skinned group, the local red variety has the highest amount of total and mono anthocyanins.

*Cyanidin derivatives*: Cyanidin 3-p-coumaroyl rutinoside-5-glucoside was tentatively annotated by Oertel et al. [9] for the first time. In our data, a variable accumulation pattern was observed within the red-skinned group, in which the local red variety has the highest amount of this compound. Whereas the concentration of the cyanidin derivative is higher in purple-fleshed varieties than the red-skinned group, a lower variability was observed within the purple group.

*Malvidin derivatives*: Several authors [10,23,26] reported that Violette is characterized by the highest levels of malvidin derivatives [23]. Our results (Figure 6) are in agreement with the literature [40], indeed, the Vitelotte cultivar contains the highest amount of malvidin 3-p-coumaroyl rutinoside-5-glucoside within the samples analyzed in this study.

*Pelargonidin derivatives*: our data show that the levels of acylated glycosides of pelargonidin are higher in red-skinned varieties than in purple-fleshed varieties, thus confirming that they are the most characteristic pigments in the red-skinned group [9]. The local red variety from Led has the highest levels of pelargonidin derivatives within the red-skinned group, in detail, the highest amount of pelargonidin feruloyl-xylosyl-glucosyl-galactoside is observed that was reported by Oertel et al. [9] for the first time. Lachman et al. [26] reported that pelargonidin 3-p-coumaric rutinoside-5-glucoside is the most abundant anthocyanin in potatoes and it was characteristic for red tuber tissue [9]. It is worth mentioning that the Blaue St. Galler variety shows the lowest content of pelargonidin derivatives.

*Petunidin derivatives*: petunidin derivatives are characteristic in the Blaue St. Galler variety and these results are confirmed by Lachman et al. [26]. In contrast the lowest amount of petunidin 3-p-coumaroylrutinoside-5-glucoside is observed in the Vitelotte cultivar, contrary to what it has been reported by Mulinacci et al. [10]. Within the red-skinned group, the highest amount of petunidin derivatives is found in the red local variety.

*Peonidin derivatives*: Blaue St. Galler contains the lowest concentration in peonidin derivatives within the purple-fleshed group, whereas the highest content is found in Violette (Saba) cultivar.

The literature reports that the most abundant anthocyanins in potates were: pelargonidin, cyanidin, delphinidin, peonidin, petunidin, and malvidin in acylated forms [7]. They are commonly acylated by caffeic, ferulic, and p-coumaric acids, for instance. Generally, the acylated anthocyanins were 98% of the total anthocyanin content in the potatoes [7] and the proportion of acylation differed between the red-skinned and purple-fleshed potato groups. Additionally, the acylated forms may be commonly linked to the rutinose, the most common glycosyl moiety, in position C3 [7], but also to other sugar moieties such as glucose, rhamnose, xylose, and arabinose. Jokioja et al. [41] described how the glycosyl moieties and the acylation influenced the chemical proprieties of the acylated anthocyanins, such as the stability of these compounds [41]. It has been reported that the different trend in the accumulation of anthocyanins in red-skinned and purple-fleshed varieties is a noteworthy factor in determining the tuber coloration patterns and the differentiation between the two pigmented groups [9]. Indeed, the proportions and the concentrations of acylated anthocyanins are variable across the pigmented potato varieties. However, all the anthocyanins under investigation has been detected in our samples, thus supporting existing results [41]. Numerous factors may influence the occurrence and accumulation of pigments in the flesh of potatoes [42]. Based on the type of acylated anthocyanin, the flesh of the pigmented tubers can be different. Indeed, Oertel et al. [9] reported that the presence of a specific anthocyanin backbone is responsible for the coloration patterns of the tubers. For example, the authors [9] reported that the red-skinned varieties contained pelargonidin derivatives, whereas the purple-fleshed cultivars are characterized by petunidin derivatives, thus confirming our results. Additionally, a negative association between pelargonidin and petunidin derivatives is found. The biosynthesis of anthocyanins is influenced by internal factors, such as enzymes and sugar levels, and by external factors, such as climatic and environmental conditions [32]. The pigments are synthesized in the cytoplasm and endoplasmic reticulum membrane, and then they are accumulated in the vacuole [32]. Wang et al. [32] reported that purple-fleshed potatoes contained higher levels of enzyme PAL (phenylalanine ammonia lyase) than yellow-fleshed potatoes, which is involved in the anthocyanin accumulation. Being the potatoes consumed cooked, the cooking process is a crucial step in evaluating the amounts of acylated anthocyanins in pigmented tubers, indeed, the cooking treatments influenced the concentration of anthocyanins [10]. The anthocyanins were soluble in water and leached into the water during the boiling [43]. Mulinacci et al. [10] reported that the level of pigments between raw and cooked potatoes remained unaltered after microwaves cooking. Nevertheless, a decrease in the total anthocyanins content was observed in boiled potatoes [10]. This discrepancy could be due to the cooking methods and the cooking time, which influence the concentration of the phytochemicals in vegetables as reported in the literature [26,42]. Thus, leading to a increasing interest in selecting the most suitable cooking approach to preserve the maximum content of healthy compounds. It has been reported that the steaming method preserves the levels of pigments in potatoes compared to boiled or untreated material [42]. Additionally, the anthocyanins were strongly dependent on the pH and temperature of water leading to a certain loss of these pigments in comparison with the raw material during cooking [10]. Fossen et al. [44] reported that the color intensity of petanin and cyanidin-3-glucoside was affected by the pH variation. A degradation in the color intensity was observed until pH 5 for both pigments, whereas in alkaline conditions (pH 8.1) the coloration had a maximum [44]. Furthermore, the stability of anthocyanins during the storage was affected by the pH value [44]. Nevertheless, the aglycones of the anthocyanins were found to be bound to sugar moieties and the anthocyanins glycoside were more stable than the monoglucosides [45]. Fleschhut et al. [45] showed that anthocyanins were less stable at 23 ${}^{{}^{\circ}}$C than at 10 ${}^{{}^{\circ}}$C and decreased in the range between pH 4–9. The stability of the pigments in the foods was an important factor for the acceptability of the consumers [30]. The properties of anthocyanins have been already studied for their anticancer, anti-diabetes, and anti-inflammatory activity. Therefore, the actual research is focused on the bioavailability of anthocyanins in humans after the consumption of food rich in such compounds [41,45]. At odds, the biological activity and healthy potential of anthocyanins is mainly evaluated *in vitro* experiments. The bioavailability of anthocyanins is affected by several factors (e.g., pH, structural stability, acylation, absorption, and metabolism) [41]. Fleschhut et al. [45] highlighted the poor bioavailability of anthocyanins in humans due to the extensive biotransformation, which occurred at the colon level. Jokioja et al. [41] reported that the acylated anthocyanins are subjected to an extensive metabolism in humans [41]. The bioavailability of acylated anthocyanins depended on their chemical proprieties, the type of anthocyanin backbone, the acyl and sugar moieties, but also the presence of other compounds in the food matrices and the cooking method had an important role in the bioavailability of acylated anthocyanins. The scientific community agreed that the acylated anthocyanins, to which di- and trisaccharides are bound, are more stable compared to nonacylated anthocyanins or acylated anthocyanins with a monosaccharide conjugate [41]. Clinical studies were conducted on healthy, hypertensive, overweight, and obese volunteers [41] evidenced that the extracts of pigmented potatoes had an important role in avoiding the increase of both postprandial glucose and insulinemia [41]. Additionally, it has been proved that acylated anthocyanins had effects on postprandial inflammation, which caused by a high assumption of carbohydrates and fats, by controlling the postprandial glucose fluctuation. Therefore, the selection of pigmented tubers, which are characterized by a high amount of different types of acylated anthocyanins, is proposed for human diet. Additionally, Moser et al. [46] showed that the purple-fleshed potatoes inhibited the glucose transportation in Caco-2 intestinal cells *in vitro*, thus highlighting the positive effects on glycemia and postprandial insulinemia [7,41].The anthocyanins are cleaved by enzymes of the gut bacteria into aglycon and phenolic acids supposing that the healthy effect of anthocyanins in humans is related to their metabolites [47]. Our results contribute to a better understanding of the metaboloma of eight pigmented potato varieties rich in health-promoting compounds to encourage the consumption of pigmented potatoes rich in different types of anthocyanins.

### 3.3. Identification of the Most Valuable Potato Varieties for Human Diets by Comparing the Different Metabolomes

**Figure 7:**
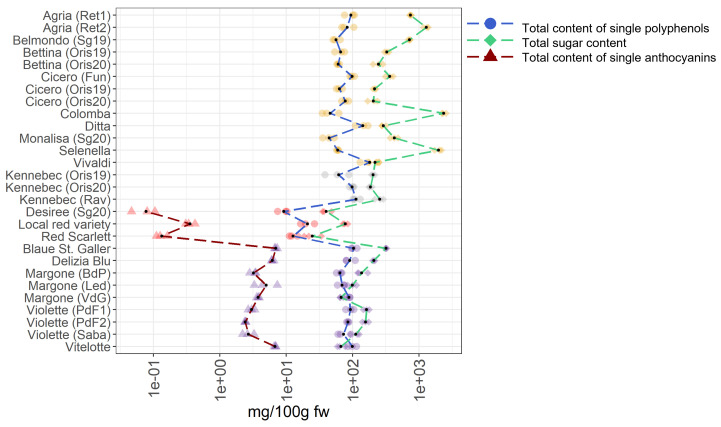
Total content of single polyphenols, total content of sugars, and total content of single anthocyanins in 28 potato varieties organized in four groups (yellow-, white-fleshed, red-skinned, and purple-fleshed); three biological replicates for each sample were analyzed. The black points represented the averages calculated on three biological replicates. The long dash lines showed the concentration trends amongst the potato groups. The concentrations were transformed in a log-10-scale. fw = fresh weight. For more detailed information about potato varieties see Table 1.

A survey is conducted to identify the most favorable cultivars for human consumption by comparing the different metabolomes investigated. This aims to point consumers towards potato varieties characterized by a balanced content in single polyphenols, sugars, and single anthocyanins, including the acylated anthocyanins. For this claim, all single compounds, namely polyphenols (reported as total content of single polyphenols), sugars (reported as total sugar content), and single anthocyanins (reported as total content of single anthocyanins) were summarized for each potato sample. The summarized data are provided in the Tables S3 and S4 and they are graphically reported in Figure 7.

In the case of total content of single polyphenols, the most noticeable varieties are Vivaldi, Ditta, and Kennebec (Rav) showing concentrations of 180.17 mg/100 g fw, 142.42 mg/100 g fw, and 112.99 mg/100 g fw, respectively, that break the trend in the total content of polyphenols compared to other varieties. Notably, these varieties are belonging to the yellow- and white fleshed groups, highlighting that these groups are rich in polyphenols. Within the red-skinned potato samples, the highest concentrations of single polyphenols is found in the local red variety (20.87 mg/100 g fw). Indeed, a marked fluctuation in the total content of single polyphenols within this group is observed. Concerning the purple-fleshed varieties, the Blaue St. Galler cultivar shows the highest total content of single polyphenols (103.84 mg/100 g fw), nearly compared to yellow- and white-fleshed potato samples. Interesting, the total content of single polyphenols in the purple-fleshed variety Margone from VdG, Led, and BdP, are showing notable concentrations and they are almost comparable between them, 88.14 mg/100 g fw, 69.28 mg/100 g fw, and 64.18 mg/100 g fw, respectively.

Regarding the total sugar content, the yellow-fleshed potato samples Colomba, Selenella, and Agria (Ret2), collected from different commercial retailers, show the highest values, namely 2,354.93 mg/100 g fw, 1,978.29 mg/100 g fw, and 1,287,86 mg/100 g fw, respectively. Notably, the total sugar content in these cultivars really stand out amongst all the samples studied and a high variability within the yellow-fleshed samples is observed, suggesting that potatoes from retailer have a higher sugar content than regionally produced ones. Indeed, the lowest total sugar content in this group is found in Ditta (290.02 mg/100 g fw) and Vivaldi (218.30 mg/100 g fw), both from local production. At odds, the total sugar content in the white-fleshed potato samples is comparable across them probably why only one variety (Kennebec) is collected in different locations and years, and a flat zone in the green dash line representing the total sugar content in Figure 7 is visible. On the contrary, in the pigmented samples, the total sugar content is significantly lower than in non-pigmented potatoes (Table S3). Within the red-skinned samples, which are all locally produced, the red local variety breaks the trend of the total sugar content due to the highest amount (77.26 mg/100 g fw), followed by the Desiree variety (40.10 mg/100 g fw) and the Red Scarlett variety (24.77 mg/100 g fw). Within the purple-fleshed potatoes, the highest sugar value in the Blaue St. Galle potato sample is found (319.00 mg/100 g fw), indeed, its content really stands out amongst all pigmented varieties (Figure 7). The most noticeable varieties in this group are Margone from Led and VdG, with a total sugar content of 99.53 mg/100 g fw and 67.27 mg/100 g fw, respectively, and Vitelotte with 66.77 mg/100 g fw.

Concerning the total content of single anthocyanins, the local red variety shows the highest value with 0.36 mg/100 g fw in the red-skinned potato group. As expected, a noteworthy increment in the trend of the total content of single anthocyanins in the purple-fleshed samples is observed (Figure 7). The variety of Margone, from Led, BdP, and VdG shows remarkable amounts of 4.99 mg/100 g fw, 3.22 mg/100 g fw, and 3.76 mg/100 g fw, respectively. The highest value in the total content of single anthocyanins is found in Blaue St. Galler (6.94 mg/100 g fw), which is locally produced, and is followed by the most famous and commercialized Vitelotte cultivar (6.78 mg/100 g fw). Indeed, the values of Blaue St. Galler and Vitelotte stand out within the purple-fleshed group (Figure 7). In summary, within non-pigmented potatoes, the most promising varieties are Ditta and Vivaldi due to the lowest total sugar content and remarkable values in the total content of single polyphenols. Concerning the red-skinned potato group, a noticeable total sugar content in the local red variety is well-counterbalanced by very high values in total content of single polyphenols and single anthocyanins. On the contrary, the total content of single polyphenols found in the purple-fleshed varieties Margone, collected in BdP, and Vitelotte is lower than the total sugar content in the same samples.

Our findings show that pigmented potatoes are characterized by a lower amount of sugars than non-pigmented potatoes, and by a higher content in single anthocyanins, and that locally produced varieties are the most promising ones for consumers.

## 4. Conclusions

In the habitual diet of Italian people, the presence of tubers with different skin and flesh colors is continuously increasing, thus stimulating the interest of researchers in evaluating their potential role in the human diet. In the present study, the metaboloma of four macro potato groups, that are yellow-, white-fleshed (non-pigmented), red-skinned, and purple-fleshed (pigmented) potatoes, was characterized by analyzing the antioxidant proprieties and the polyphenolic and sugar profiles. Furthermore, the anthocyanin profile in the pigmented potato samples was elucidated. The aims were to identify specific potato cultivars rich in health-promoting compounds for the human diet and to promote the valorization of locally cultivated potato varieties. The results showed that the yellow- and white-fleshed potato samples, were characterized by low antioxidant proprieties, on the contrary, they had the highest content in single polyphenols and sugars. The red-skinned potato varieties showed similarities in the antioxidant properties compared to non-pigmented samples, however, the red-skinned samples were characterized by the lowest content of single polyphenols and sugars and showed a low anthocyanin content. The purple-fleshed varieties stand out because of the highest antioxidant activity and highest content of anthocyanins, moreover, they showed remarkable content of single polyphenols and a reasonable sugar content. Within the non-pigmented potatoes, the most promising varieties were the locally produced Ditta (BZ, Italy) and Vivaldi (TN, Italy) having the lowest amount of sugars and a remarkable content of polyphenols, followed by the varieties Bettina (BZ, Italy), Cicero (BZ, Italy), and Kennebec (BZ, Italy). Within the red-skinned group, the local red variety collected in Led (TN, Italy) showed the highest content in single polyphenols, sugars, and anthocyanins. The potatoes belonging to the purple-fleshed group are the most promising for human diet. A special interest was driven towards to the cultivar of Margone from local production. In particular, the best-balanced concentration in phytochemicals was found in the Margone sample from VdG (TN, Italy), characterized by high antioxidant propriety and polyphenolic content, a medium-low concentration of sugars, and an appreciable amount of anthocyanins, followed by the Margone samples from BdP (TN, Italy) and Led (TN, Italy). The Blaue St. Galler cultivar, collected from Val Pusteria (BZ, Italy), had the highest sugar content within the purple-fleshed group, however, this variety was well-counterbalanced by a moderate amount of polyphenols and a considerable amount of anthocyanins. Additionally, a good balance in the antioxidant propriety and in the concentration of polyphenols, sugars, and anthocyanins was found in the well-known, purple-fleshed variety cultivar Vitelotte (VT, Italy). In conclusion, the present study showed that locally produced potatoes, in particular the purple-fleshed varieties, contained the highest amounts of health promoting compounds, thus highlighting the importance of locally cultivated potatoes for human diet.

## Figures and Tables

**Table 1:** List of cultivars used in this study. The potato varieties are ordered by the group. The “sample ID” gathers the abbreviations used in the Figures 3–7.

Sample Number	Variety	Origin	Year	Sample ID	Group	Pigmentation	Skin Color	Pulp Color	Maturity	Shape
1	Belmondo	San Genesio (BZ)	2019	Belmondo (Sg19)	Yellow	Non-pigmented	Yellowish	Yellow	Early	Round-oval
2	Monalisa	San Genesio (BZ)	2020	Monalisa (Sg20)	Yellow	Non-pigmented	Golden-yellow	Golden-yellow	Medium early	Oval
3 4	Bettina	Oris (BZ)	2019 2020	Bettina (Sg19)	Yellow	Non-pigmented	Yellow	Light yellow	Medium early	Oval
5 6 7	Cicero	Oris (BZ) Oris (BZ) Funes (BZ)	2019 2020 2020	Cicero (Oris19) Cicero (Oris20) Cicero (Fun)	Yellow	Non-pigmented	Yellow	Pale Yellow	Medium early	Oval
8 9	Agria (Patata di Montagna)	Ret1 Ret2	2019	Agria (Ret1) Agria (Ret2)	Yellow	Non-pigmented	Yellow	Yellow	Early	Long oval
10	Selenella	Ret3	2019	Selenella	Yellow	Non-pigmented	Yellow	Yellow	Early	Oval
11	Colomba	Ret4	2019	Colomba	Yellow	Non-pigmented	Yellow	Yellow	Early	Round oval
12	Ditta	Merano	2020	Ditta	Yellow	Non-pigmented	Yellow	Light yellow	Early	Long oval
13	Vivaldi	Calceranica al lago (TN)	2020	Vivaldi	Yellow	Non-pigmented	Yellow	Light yellow	Medium early	Oval
14 15 16	Kennebec	Oris (BZ) Oris (BZ) Ravina (TN)	2019 2020 2020	Kennebec (Oris19) Kennebec (Oris20) Kennebec (Rav)	White	Non-pigmented	Yellow	White	Early	Oval
14	Red Scarlett	Calceranica al lago(TN)	2020	Red Scarlett	Red	Pigmented	Red	Light yellow	Medium early	Long oval
18	Desire	San Genesio (BZ)	2020	Desire (Sg20)	Red	Pigmented	Red	Light yellow	Medium late	Oval
19	Local red variety	Val di Ledro (TN)	2020	Local red variety	Red	Pigmented	Red	Light yellow	Medium late	Round oval
20 21 22	Margone	Val di Ledro (TN) Val di Gresta (TN) Baselga di Pinè (TN)	2020	Margone (Led) Margone (VdG) Margone (BdP)	Purple	Pigmented	Dark violet	Dark violet	Very late	Round oval
23 24 25	Violette	Sabaudia (LT) Piana del Fucino1 (AG) Piana del Fucino2 (AG)	2020	Violette (Saba) Violette (PdF1) Violette (Pdf2)	Purple	Pigmented	Dark violet	Dark violet	Very late	Long irregular
26	Vitellotte	Grotte di Castro (VT)	2020	Vitellotte	Purple	Pigmented	Dark violet	Dark violet	Very late	Long irregular
27	Delizia Blu	Grotte di Castro (VT)	2020	Delizia Blu	Purple	Pigmented	Dark violet	Dark violet	Very late	Round oval
28	Blaue St. Galler	Val Pusteria (BZ)	2020	Blaue St. Galler	Purple	Pigmented	Dark violet	Dark violet	Very late	Long irregular

**Table 2:** List of datasets used for the data fusion approach based on JIVE. Each dataset was identified by a dataset ID and gathered a specific number of analytes. The analytical methods were reported. More detailed information regarding the quantitative data, which composed the datasets, can be found in Table S3 (AA, Poly, Sug) and Table S4 (Anthocyanins).

Dataset	Dataset ID	N. of Analytes	Analytical Methods	JIVE Model
Antioxidants Total polyphenolic content	AA	3	Spectrophotometric assays (FRAP, ABTS, FC)	yes
Polyphenols	Poly	15	UHPLC-QqQ-MS/MS	yes
Sugar	Sug	7	IC-HPAE-PAD	yes
Anthocyanins	Anthocyanins	22	UHPLC-QqQ-MS/MS	no

## Data Availability

The data presented in this study are available in article and Supplementary.

## Supplementary Materials

The following supporting information can be downloaded at: https://www.mdpi.com/article/10.3390/foods11121708/s1, Table S1: Polyphenol-Compounds analyzed with UHPLC-QqQ-MS/MS in this study, the corresponding compound class, compound number, compound name, retention time, ESI polarity, molecular weight, precursors, products, collision energy, RF lens, regression parameters and linearity range are reported. Table S2: Anthocyanins-Compounds analyzed with UHPLC-QqQ-MS/MS in this study, the corresponding compound name, compound name, acronym, retention time, transitions, declustering potential, entrance potential, collision energy, collision cell exit potential, the regression (r2) and slope of the cyanidin-3-glucoside are reported. Table S3: List of the potato samples (column A), varieties (column B), sugars (column D-I; dataset sug), antioxidant and total polyphenolic content (column J-L; dataset AA), polyphenolic compounds (column M-AA; dataset poly) quantified in the potato samples included in this study. Results are expressed as mg/100g fw. In column AB (total sugar content) the sum of all sugars are reported, in column AC (average of total sugar content) the average content of total sugars of the three biological replicates for each sample are reported, in column AD (total content of single polyphenols) the sum of all single polyphenols are reported, in column AE (average of total content of single polyphenols) the average content of the total single polyphenols of the three biological replicates for each sample are reported. Table S4: List of the potato samples (column A), varieties (column B), Total Monomeric Content and Total Anthocyanins Content (Anto mono and Anto tot) (column D–E) and single anthocyanins (column F-AA) quantified in the potato samples included in this study. Results are expressed as mg/100g fw. Acronym of anthocyanin-compounds are reported Table S2. In column AB (total content of single anthocyanins) the sum of single anthocyanins are reported, in column AC (average of total content of single anthocyanins) the average content of single anthocyanins of the three biological replicates for each sample are reported. Table S5: Compounds analyzed with ion chromatograph (IC) with pulsed amperometric detection (HPAE-PAD) in this study, the corresponding compound number, compound name, retention time, molecular weight, regression parameters and linearity range are reported.

## Abbreviations

AA, antioxidants; ABTS, 2,2′-Azino-bis-(3-ethylbenzothiazoline-6-sulfonic acid; ADA, American Diabetes Association; Anto mono, Total Monomeric Content; Anto tot, Total Anthocyanins Content; a.s.l, above sea level; CDS, Chromatography Data System; ESI, Electrospray ionization; FAO, Food and Agriculture Organization; FC, Folin–Ciocalteu; FRAP, Ferric Reducing Antioxidant Power Assay; fw, fresh weight; GI, glycaemic index; HPAE-PAD, High-Performance Anion-Exchange chromatography with Pulsed Amperometric Detection; IC, ion chromatograph; JIVE, Joint and Individual Variation Explained; LC-MS, liquid chromatography-mass spectrometry; LOD, limit of detection; LOQ, limit of quantification; LV, latent variable; MRM, Multiple-reaction monitoring; MW, molecular weight; PAL, phenylalanine ammonia lyase; PCA, principal component analysis; Polyphenols, poly; PTFE, Polytetrafluoroethylene; QC, quality control sample; RT, room temperature; RSD, relative standard deviation; SCA, simultaneous component analysis; Sugars, sug; SVD, Singular Value Decomposition; TEAC, Trolox equivalent antioxidant capacity; TPC, Total Polyphenolic Content; TPTZ, 2,4,6-Tris(2-pyridyl)- s-triazine; Trolox, 6-hydroxy-2,5,7,8-tetramethylchroman-2-carboxylic acid; UHPLC-QqQ-MS/MS, high-performance liquid chromatography coupled with triple quadrupole mass spectrometry; VIP, variable importance.
